# Permanent diabetes insipidus in a patient with mesothelioma treated with immunotherapy

**DOI:** 10.20945/2359-3997000000221

**Published:** 2020-03-30

**Authors:** Lucia Brilli, Luana Calabrò, Michele Campanile, Tania Pilli, Cristina Agostinis, Alfonso Cerase, Michele Maio, Maria Grazia Castagna

**Affiliations:** 1 Unit of Endocrinology Department of Medical, Surgical and Neurological Sciences University Hospital of Siena Siena Italy Unit of Endocrinology, Department of Medical, Surgical and Neurological Sciences, University Hospital of Siena, Siena, Italy; 2 Center for Immuno-Oncology Department of Medical Oncology University Hospital of Siena Siena Italy Medical Oncology and Immunotherapy, Center for Immuno-Oncology, Department of Medical Oncology, University Hospital of Siena, Siena, Italy; 3 Unit of Neuroradiology “Papa Giovanni XXIII” Hospital Bergamo Italy Unit of Neuroradiology, “Papa Giovanni XXIII” Hospital, Bergamo, Italy; 4 Unit of Neuroimaging and Neurointervention Department of Neurological and Sensorineural Sciences University Hospital of Siena Siena Italy Unit of Neuroimaging and Neurointervention, Department of Neurological and Sensorineural Sciences, University Hospital of Siena, Siena, Italy

**Keywords:** Diabetes insipidus, immunotherapy, durvalumab, tremelimumab

## Abstract

Checkpoint inhibitors have substantially improved the prognosis for patients with advanced malignancy. Treatment with immunomodulants has the ability to reactivate the immune system against tumor cells, but can also trigger the development of immune-related adverse events that reflects a loss of tolerance of the immune system for self-antigens. Regarding the endocrine system, thyroid and pituitary are the most frequent glands involved; in particular hypophysitis is commonly observed with anti-CTLA4 with a variable impaired anterior pituitary dysfunction (mainly ACTH and TSH dysregulation) while a posterior pituitary dysfunction has been rarely described. A 68-year-old man with a diagnosis of metastatic mesothelioma started in September 2016 first-line treatment with tremelimumab and durvalumab. After 3 cycles he presented sudden onset of polydipsia and polyuria without other symptoms. Diagnostic work-up, including a water deprivation test, established a diagnosis of central diabetes insipidus. Patient started sublingual desmopressin 60 mcg three times a day, that was subsequently increased up to 480 mcg/die. At magnetic resonance imaging the posterior lobe of pituitary gland did not show high signal intensity on T1-weighted images. After regression of diabetes insipidus symptoms under desmopressin, patient restarted cancer treatment and received additional 10 doses without worsening of endocrinological toxicity or further treatment-related toxicities, maintaining the same desmopressin dosage. Posterior pituitary dysfunction has been rarely observed in patients treated with immunomodulants. To our knowledge, this is the first observation of permanent central diabetes insipidus in patients treated with combined immune checkpoint inhibitors (tremelimumab and durvalumab).

## INTRODUCTION

Targeting immune checkpoint inhibitors has become a cornerstone in the treatment of metastatic melanoma and an effective treatment option for other solid tumor types. These drugs are also under investigation for almost all tumor types (1).

The upregulation of the immune system, while enhancing antitumor response, may also induce immune-related adverse events (AEs) and can affect one or more organs. The endocrine system is one of the most frequent involved (2,3). The management of these AEs requires a multidisciplinary approach involving not only oncologists, but also other specialists, to ensure a prompt diagnosis and an optimal management.

Hypophysitis represents the most frequent endocrinopathy with anti-cytotoxic T lymphocyte antigen (CTLA-4) treatment (up to 15-20%) (4-7) while the prevalence is extremely lower (<1%) with anti-PD-1 and anti-PD-L1 therapy (3). Nowadays combined treatments (CTLA-4+PD-1/PD-L1) are currently under investigation in many solid cancers. Under these treatments, toxicities are more common and an increased prevalence of endocrinopathies, including hypophysitis, could be expected in the next future.

Hypophysitis usually causes a damage of the anterior pituitary gland; ACTH secretion is frequently, and almost permanently, impaired while other anterior pituitary hormone secretions, although reduced at the diagnosis of the deficiency, are commonly restored during follow-up. Involvement of posterior pituitary gland is estremely rare in hypophysitis due to immunotherapy (2,8,9) and, to our knowledge, it was never reported as both isolated and mostly permanent.

In the present report we describe a case of central diabetes insipidus (DI) occurred in a patient with metastatic mesothelioma treated with the anti-CTLA-4 tremelimumab and the anti-PD-L1 durvalumab. An informed consent was obtained.

A 68-year-old Caucasian man was admitted to the hospital in March 2017 because of polydipsia and polyuria. His family history was unremarkable. He was exposed for more than 10 years to asbestos and in 2016 an histological diagnosis of mesothelioma was made. The patient refused standard treatment (platinum-pemetrexed) and, in September 2016, started first line treatment with tremelimumab 1 mg/kg iv d1, q4 weeks (wks) for 4 cycles and durvalumab 20mg/kg iv d1 q4 wks for 13 cycles within the NIBIT-MESO-1 study.

**Figure 1:**
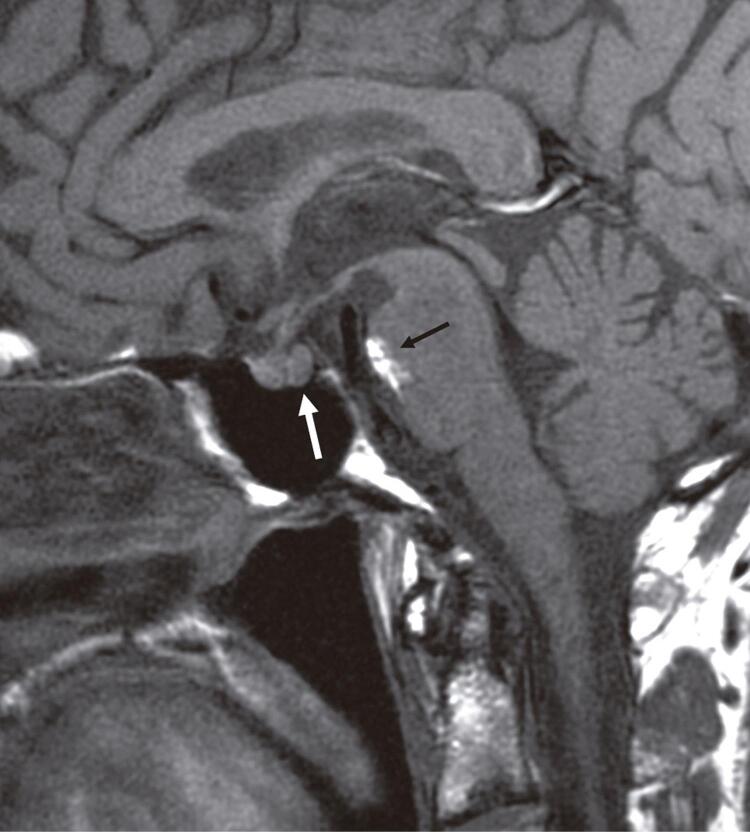
T1-weighted magnetic resonance sagittal image shows absence of high signal intensity of the posterior pituitary lobe (white arrow). In the anterior pons, note a lesion consistent with dermoid cyst (black arrow), as an incidental finding.

After the 3^rd^ cycle (at week 12) the patient achieved a partial response on CT scan. At week 24 the patient complained about a sudden onset of polydipsia associated with polyuria and nycturia. No history of headache or fatigue was reported. At physical examination, the patient was 180 cm tall and weighed 72 kg, and body temperature, blood pressure, and oxygen saturation by pulse oximeter on room air were 35.6°C, 120/70 mmHg, and 98%, respectively. The 24 hours urine volume was large (4000 mL/day) and unbalanced with respect to the liquid intake (3200 mL/day). Laboratory findings at the baseline showed normal levels of serum sodium, plasma osmolality (P_osm_) and urinary specific gravity test (urine osmolality is not available at our hospital). Thyroid function was normal as well as the anterior pituitary function. Pituitary gland reserve was tested showing a normal response of all hormones. Pituitary antibodies, detected by indirect immunofluorescence assay (Euroimmun, Lubecca, Germany) using as substrate a monkey pituitary gland, were negative. Cancer treatment was temporary withdrawn and a water deprivation test was performed: after 10 hours sodium levels increased up to 150 mEq/L and 2 hours later there was a further increase up to 152 mEq/L along with a weight loss of 3.8% and a P_osm_ of 299 mOsm/L (Table 1). Based on these findings, the test was stopped and a desmopressin stimulation test (2 mcg i.m.) was performed. After that, serum sodium and P_osm_ returned to normal values (Table 2). Magnetic resonance imaging (MRI) of the head, brain, pituitary and diencephalic region did not show signs of intracranial metastases. The posterior lobe of pituitary gland (Figure 1) did not show high signal intensity on T1-weighted images (no previous sellar imaging before cancer treatment is available).

**Table 1:** Laboratory findings during water deprivation test

Time	Weight (kg)	Weight loss (%)	Urinary volume (mL)	Urinary specific gravity test (n.v. 1005-1025)	Na (mEq/L) (n.v. 132-148)	K (mEq/L) (n.v. 3.5-5.5)	Plasma osmolarity (mOsm/L) (n.v. 275-295)
0:00	73,4						
8:00	71,2	3	1900	1005	149	3,6	293
10:00	71	3,27	200	1005	150	3,9	296
12:00	70,6	3,81	200	1010	152	3,7	299

**Table 2:** laboratory findings after desmopressin stimulation test

Time	Urinary specific gravity test (n.v. 1005-1025)	Na (mEq/L) (n.v. 132-148)	K (mEq/L) (n.v. 3.5-5.5)	Plasma osmolarity (mOsm/L) (n.v. 275-295)
After 3 hours from Emosint 2 mcg	1010	147	3,3	290
After 5 hours from Emosint 2 mcg	1005	145	3,6	285
After 12 hours from Emosint 2 mcg	1005	147	3,6	289

A diagnosis of central DI was made and the patient was started on treatment with desmopressin (DDAVP) tablets 60 mcg three times a day.

After 10 days, laboratory findings showed hypernatremia (150 mEq/l) and a P_osm_ at the upper limit of the range, therefore the DDAVP dosage was gradually increased up to 480 mcg/day. After regression of DI symptoms under DDAVP, patient restarted cancer treatment (withdrawal for 28 days) and received additional 10 doses without worsening of endocrinological toxicity or further treatment-related toxicities. On April 2018 cancer treatment was permanently discontinued due to a progressive disease and patient was still continuing desmopressin at the last follow-up visit performed after further 6 months.

Central DI is defined as a decreased secretion of arginine vasopressin (AVP) leading to a high urine output (>3L/24 hours). Polyuria becomes clinically apparent when 80% of AVP-secreting neurons are destroyed. Central DI is an uncommon condition, usually caused by lesions in supraoptic and paraventricular nuclei. DI has also been identified in a variety of pituitary conditions such as inflammatory disease, traumatic injury in addition to an idiopathic form. Central DI seems to be an extremely rare side effect of immunotherapy and, to our knowledge, this is the first reported case of isolated and permanent DI in a patient treated on a combination regimen with the anti-CTLA-4 inhibitor, tremelimumab and the anti-PD-L1 inhibitor, durvalumab. In our case DI did not recover during the temporary discontinuation of immunotherapy (28 days), on the contrary, in this time frame, patient needed a progressive increase of desmopressin dose up to 480 mg.

Only three cases of central DI have been previously described: two patients treated with the anti-CTLA-4 ipilimumab (8,10) and one patient treated with the anti-PD-L1 avelumab (9), with or without anterior pituitary damage. In two cases DI was transient recovering 3 days and 6 weeks after starting corticosteroids (8,9). In the last case desmopressin was still continuing after 4 months but no data in a longer follow-up (10). In our patient the irreversibility of the DI may be related to the combined treatment (anti-CTLA-4+-PDL-1) that could cause more severe immune-related AEs than monotherapy alone; we did not administer corticosteroids, since according to the NCCN guidelines, high dose steroid treatment is suggested only in the presence of severe acute symptoms (e.g. headache, fever) (11).

Although corticosteroids, if administered, could have affected the clinical course of the DI in our patient, we believe that the combined treatment induced a such extensive damage that the posterior pituitary gland could have a very little chance to recover.

The etiology of infundibulo-neurohypophysitis, as well as in case of the anterior hypophysitis, remains unknown. The diagnosis of central DI is usually made within the first 12 weeks from initiation of treatment with immunomodulants; in our case the disease onset of DI occurred a little later than expected. In our patient, MRI did not show signs of intracranial metastases neither high signal intensity on T1-weighted images of the posterior pituitary gland. In central DI, MRI usually shows absence of T1 hyperintensity generally observed in the posterior pituitary gland, i.e. the so-called pituitary bright spot, thought to result from the T1-shortening effect of stored vasopressin (12). This finding may be seen in conditions resulting in depletion of vasopressin granules and has been reported in 25 to 100% of patients with central DI. However, pituitary bright spot may be absent in up to 48% of normal subjects, especially in adulthood and elderly (10). Therefore in case of DI, without involvement of anterior pituitary lobe, MRI images alone cannot be sufficient and both clinical presentation and biochemical parameters become essential to make a correct diagnosis.

In view of the rarity of this condition, DI should be investigated only in the presence of symptoms such as polyuria and polydipsia while anterior pituitary function (mainly pituitary-thyroid and pituitary-adrenal axes) should be periodically evaluated in patients treated with check-point(s) blocking therapies as recommended by several authors. Moreover, a periodic screening of the pituitary function seems to be mandatory in case of combined treatments and the need of a multidisciplinary approach for the diagnosis of DI, involving also endocrinologists and neuroradiologists should be emphasized.
